# Gene profiling and bioinformatics analyses reveal time course differential gene expression in surgically resected colorectal tissues

**DOI:** 10.3892/or.2014.3053

**Published:** 2014-02-24

**Authors:** AYA YAMAGISHI, SATOSHI MATSUMOTO, ATSUSHI WATANABE, YOSHIAKI MIZUGUCHI, KEISUKE HARA, HAYATO KAN, TAKESHI YAMADA, MICHIHIRO KOIZUMI, SEIICHI SHINJI, AKIHISA MATSUDA, JUNPEI SASAKI, TAKASHI SHIMADA, EIJI UCHIDA

**Affiliations:** 1 Surgery for Organ Function and Biological Regulation, Graduate School of Medicine, Nippon Medical School, Tokyo 113-8603, Japan; 2 Department of Biochemistry and Molecular Biology, Nippon Medical School, Tokyo 113-8603, Japan

**Keywords:** colorectal cancer, RNA degradation, clustering, bio- informatics, microarray

## Abstract

It has previously been reported that gene profiles in surgically-resected colorectal cancer tissues are altered over time possibly due to the different tissue-acquisition methods and sample extraction timing that were used. However, the changes that occur are still not clearly understood. In the present study, time-dependent changes in gene expression profiling in colorectal surgical specimens were analyzed. Normal and tumor tissues at several time-points (0, 30, 60 and 120 min) were extracted, and RNA quality, microarray experiments, quantitative PCR and bioinformatics clustering were performed. Although RNA integrity was preserved 2 h after resection, inherent increased/decreased gene expression was observed from 30–120 min in approximately 10% of genes. Bioinformatics clustering could not distinguish case-by-case, probably due to gene profiling changes. Irregular changes in gene expression after surgical resection were found, which could be a crucial confounding factor for quantitative analyses.

## Introduction

Every year, more than one million individuals worldwide develop colorectal cancer for which the disease-specific mortality rate is nearly 33% in the developed world (1). Recent clinical trials of adjuvant chemotherapy following surgical resection have shown significant survival benefits in patients with locally advanced colon cancer (2). In addition to the variety of clinical and pathological risk factors, various prognostic genetic and molecular biomarkers have been investigated to assess individual therapeutic intervention (3,4).

Although several prognostic molecular markers have been reported, the results have been contradictory or inconclusive (5,6). Recent microarray analyses provided simultaneous whole-genome screening, and recent studies identified a set of specific gene expression profiles that could predict the risk of recurrence using tests such as the Oncotype DX Colon Cancer Assay (7) and ColoPrint (8). Notably, compared with these prognostic gene-profiling series, the gene sets differ considerably, with little overlap. The lack of concordant genes could be related to several issues, including different microarray platforms and the type of samples selected for analyses. RNA instability is well known, and gene expression in tissue biosamples can be changed by various factors, including surgical manipulation with vessel occlusion, warm *ex vivo* ischemia between surgical extirpation and sample freezing, the preservation method used, and the length of storage time (9,10). However, how the expression profiles of genes change in surgically resected specimens remains unclear. Some stress-responsive genes, such as FBJ murine osteosarcoma viral oncogene homolog (FOS) and jun B proto-oncogene (JUNB), may actively increase their expression levels in hypoxic circumstances, whereas others, including keratine 20 (KRT20) and cyclin A, may decrease their expression levels in such environments (11,12). These can be critical confounding factors for quantitative comparisons among studies. These observations led us to hypothesize that genes profiled in resected specimens are altered in a time-dependent manner. In the present study, we investigated time-dependent changes in gene profiling as well as in RNA quality in surgically resected colorectal tumor tissues that were obtained using standard surgical procedures.

## Materials and methods

### Patients, sample collection and RNA isolation

Tumor and normal tissues from 18 patients with locally advanced colorectal cancer who underwent curative surgery at the Department of Surgery, Nippon Medical School Hospital, from June 2011 to May 2013 were analyzed. The patients all provided written informed consent. The study protocol was approved by the Gene Institutional Review Board of Nippon Medical School.

Normal and tumor samples were extracted directly from each resected tissue punctually at 0, 30, 60 and 120 min after their removal in the operating room, following routine tissue handling protocols of our department. Inclusion criteria were stage II/III (13) colorectal cancer patients with tumors >3 cm in diameter. Using a surgical procedure with a no-touch isolation technique (14) and preserving the blood supply from the marginal artery just before resection may have minimized the *in vivo* ischemic effect. The time of completion of the colorectal resection was set to 0 min. Tumor tissue samples were obtained from non-ulcerated but elevated lesions, and normal tissue samples were obtained from normal mucosa >10 cm away from the tumor margin. Each sample, ~5 mm^3^ in size, was placed immediately into 5 ml of RNAlater RNA Stabilization reagent (Ambion, Inc., Austin, TX, USA). After stabilization overnight at 4°C, all samples were stored at −20°C until RNA extraction.

Following homogenization using Precellys 24 (Bertin Technologies, Saint-Quentin-en-Yvelines Cedex, France) in microcentrifuge tubes, total RNA was extracted using RNeasy kits (Qiagen, Valencia, CA, USA) according to the manufacturer’s instructions. Total RNA concentrations (ng/μl) determined at absorbances 260 nm (A260) and 280 nm (A280) using a NanoDrop ND-2000 spectrophotometer (Thermo Fisher Scientific, Wilmington, DE, USA) were used to calculate total RNA yield (μg).

### RNA integrity evaluation

RNA integrity was analyzed using an Agilent 2100 Bioanalyzer RNA 6000 NanoLabChip (Agilent Technologies, Palo Alto, CA, USA) to produce an electrophoresis trace, from which the RNA integrity number (RIN) (15) was calculated using 2100 Expert Software (Agilent Technologies). RIN is the new standard in RNA integrity assessment and the best predictor of microarray quality (16). Total RNA degradation was calculated automatically from the RIN score based on decreases in 28S and 18S ribosomal RNA peak areas (peaks correspond to degradation fragments). RIN values range from 10 for intact RNA to 1 for totally degraded RNA (15).

### Microarray hybridization, image acquisition and data analyses

Total RNA (200 ng) was converted into labeled complementary RNA (cRNA) with nucleotides coupled to cyanine 3-CTP (Cy3) using the Agilent Low Input Quick Amp (one-color) labeling kit (Agilent Technologies). Then, 1.65 μg of Cy3-labeled cRNA was hybridized to an Agilent Human GE 4x44K v2 Microarray kit for 17 h at 65°C with SurePrint technology. The Human Microarray carries 34,127 probes to more than 21,756 human Entrez genes. Each array was scanned using the Agilent G2565CA Microarray Scanner. Microarray data sets were normalized by GeneSpring GX software (version 11.5; Agilent Technologies) using the Agilent FE one-color scenario. Spots that did not pass quality control procedures were flagged as ‘Not Detected’ and ‘Compromised’. Data were normalized using the quantile method.

The MIAME-compliant microarray data are available at http://www.ncbi.nlm.nih.gov.geo/ under accession number GEO: GSE50746.

### Quantitative RT-PCR

TaqMan Gene Expression assays (Applied Biosystems, Foster City, CA, USA) were used to validate the microarray results and gene expression levels. We selected the following four genes for validation: interleukin 8 (IL8, Hs01553824_g1), granzyme B (also known as granzyme 2 or cytotoxic T-lymphocyte-associated esterase 1; GZMB, Hs00188051_m1), carbonic anhydrase II (CA2, Hs00163869_m1), and regenerating islet-derived 3 α (REG3A, Hs010555563_gH). IL8, GZMB and CA2 were reported as prognostic molecular biomarkers in colorectal cancer (3,5,6,17) and the expression level of REG3A decreased by >2-fold at 120 min (compared with its expression at 0 min) in microarray-examined cases. First-strand complementary DNA (cDNA) was synthesized from 4 μg of total RNA using a SuperScript II First-Strand Synthesis System (Invitrogen, Carlsbad, CA, USA) according to the manufacturer’s instructions. Quantitative reverse transcription PCR (qRT-PCR) was performed using 15 ng of cDNA and the TaqMan Universal PCR Master Mix (Applied Biosystems) with gene-specific primers on an Applied Biosystems Prism 7500 Fast sequence detector (Applied Biosystems). All assays were performed in triplicate, and the results were normalized against glyceraldehyde-3-phosphate dehydrogenase (GAPDH) (Hs99999905_m1). The standard curve-based method was used to calculate relative expressions.

### Statistical analyses

The RIN score results in the present study were analyzed using SPSS Statistics Base 20 (IBM, Chicago, IL, USA), with data presented as mean ± SD. Paired t-tests were used to compare values of two related groups. Differences were considered statistically significant if P<0.05.

## Results

### Clinical and pathological characteristics of the study population

The clinical and pathological characteristics of the 18 patients are shown in Table I. The mean age of the patients was 72.4 years (range, 53–85 years), and the male to female ratio was 10:8. The mean diameter size of the tumors was 47.8 mm. Pathologically, half of the tumors were well-differentiated adenocarcinoma, and half were moderately differentiated adenocarcinoma. Ten patients had stage II disease and eight patients had stage III disease. None of the patients had distant metastases.

### RNA integrity is preserved for 120 min in resected colorectal specimens

**Figure 1:**
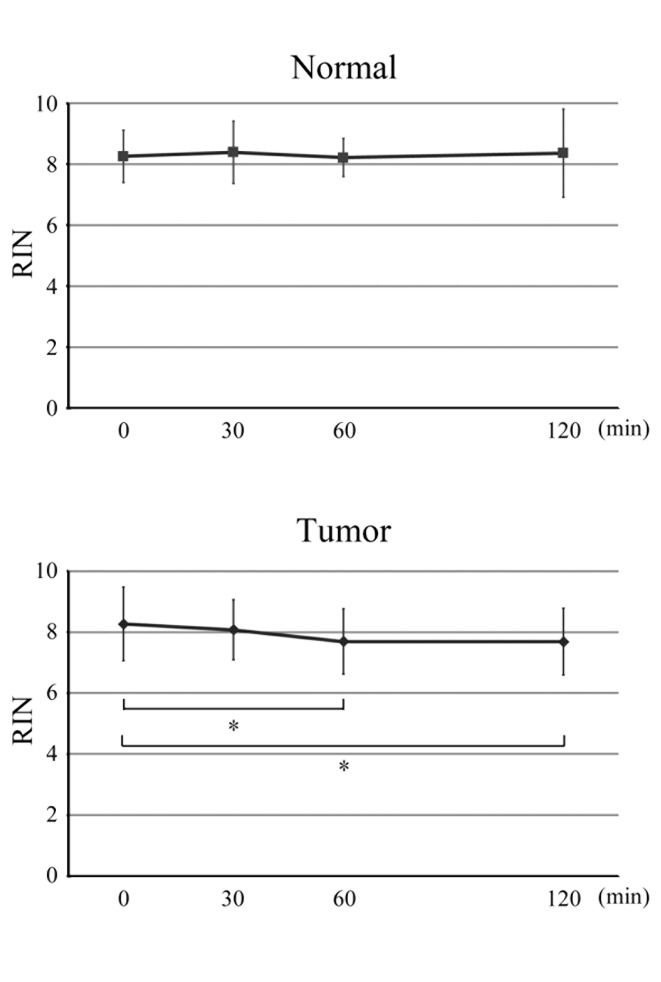
RNA integrity at several time-points in surgically resected colorectal samples. The RNA integrity number (RIN) at 0, 30, 60 and 120 min is shown for normal (upper) and tumor (lower) samples from the 18 patients. The RIN is maintained over the 120 min. ^*^P<0.05 compared with the 0-min time-point. Each bar represents the mean ± SD of all samples.

The most common problem for quantitative analyses using surgically resected tissues is RNA degradation. Therefore, we sought to evaluate RNA degradation in all 18 cases using RIN scores. The mean RIN scores at 0, 30, 60 and 120 min in the normal and tumor samples are shown in Fig. 1. At room temperature, no statistically significant RNA degradation was found in the normal samples and, at each time-point, no significant differences in mean RIN scores between the normal and tumor samples were observed. Although in the tumor samples, mean RIN scores decreased at 60 and 120 min (P=0.029 and 0.041, respectively), which might be a source of bias in quantitative analyses, the mean RIN scores of both the normal and tumor samples were >7 at all time-points, indicating that RNA quality was maintained in all the samples and that RNA was not degraded 120 min after surgical resection.

### Microarray analyses and gene profiling at different time-points

**Figure 2:**
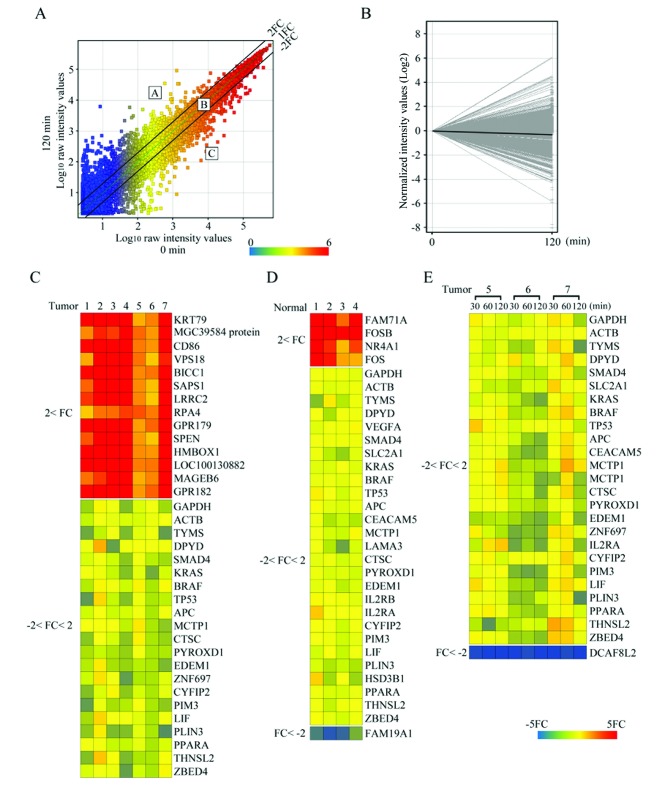
Gene profiling of surgically resected colorectal samples. (A) Representative scatter plot of changes in gene expression levels between 0 and 120 min in a tumor case. All detected tumor sample intensity values were plotted. The central diagonal lines were used to classify gene expression levels into three groups; group A, >2-fold increase in gene expression; group B, gene expression levels within a 2-fold-change; and group C, >2-fold decrease in gene expression at 120 min. (B) Expression profile plot analyses in tumor sample from representative case with high RIN values. All detected gene intensities were normalized to ‘0’ at 0 min. The gray lines join the 0 min and 120-min intensities and indicate changes in gene expression levels at the 120-min time-point. The black lines indicate alterations in GAPDH expression. (C–E) Representative heat maps of changes in gene expression levels detected in the microarray data; (C) individual genes that belonged to the same group of tumor samples in seven cases; (D) normal samples in four cases between 0 and 120 min; (E) tumor samples in three cases at the 0, 30, 60 and 120-min time-points. Note the genes in group B (−2<fold change <2) are representative.

To assess gene profiling changes among the different time-points, we comprehensively examined the microarray data. For the initial microarray analyses, we selected seven cases with high RIN values at all time-points to eliminate possible bias from RNA degradation. The expression profiles of all the samples passed the microarray quality control, and the differential expression of the genes between 0 and 120 min was examined. A representative scatter plot shows the observed changes in gene expression levels between 0 and 120 min in a tumor case (Fig. 2A). Fig. 2B shows the expression profile plot analyses for representative case with high RIN values at the 0- to 120-min time-points. In Fig. 2A, the points above the central diagonal line represent increased gene expression, and the points below the central diagonal line represent decreased gene expression at 120 min. The two parallel lines, one above and one below the central diagonal line, allowed us to classify gene expression levels into three groups: group A, a >2-fold increase in gene expression; group B, gene expression levels within a 2-fold change; and group C, a >2-fold decrease in gene expression. Group B contained most of the genes (28,055 or 88%) in the tumor samples. The mean number of probes in group A and group C was 1,964 (6.2%) and 1,862 (5.9%), respectively. Genes (n=17,537) (55% of the total) in all tumor samples from seven cases were classified in group B. These genes included some housekeeping genes, as well as genes that are important in colon cancer biology [including GAPDH, thymidylate synthase (TS) and dihydropyrimidine dehydrogenase (DPD)]; (Fig. 2C). Additionally, several colorectal biomarkers, including Kirsten rat sarcoma viral oncogene homolog (KRAS), v-raf murine sarcoma viral oncogene homolog B (BRAF) (17), TS, DPD (18), SMAD family member 4 (SMAD4) (19) and most ColoPrint reference genes (8), including multiple C2 domains, transmembrane 1 (MCTP1), cathepsin C (CTSC) and pyridine nucleotide-disulphide oxidoreductase domain 1 (PYROXD1), were in group B, indicating the reliability and reproducibility of these genes for molecular analyses in colorectal cancer. Of note, however, one ColoPrint-related gene, hydroxy-δ-5-steroid dehydrogenase, 3β- and steroid δ-isomerase 1 (HSD3B1), had a wide expression distribution among the time-points. Fifteen genes belonged to group A in all seven tumor cases. They included genes that were reported to be colorectal cancer-related genes [including CD86 molecule (CD86) (20), vacuolar protein sorting 18 homolog (VPS18) (21) and protein phosphatase 6, regulatory subunit 1 (SAPS1) (22)]. None of the genes detected in the tumor samples from all seven cases belonged to group C. A comparison of normal samples from four cases at 0 min and 120 min showed that 22,000 genes belonged to group B, 4 genes belonged to group A and 1 gene belonged to group C (Fig. 2D). To examine differential gene profiling in more detail, we selected three tumor cases that showed high RIN values at every time-point and performed additional microarray analyses at the 30- and 60-min time-points. We found that overall ~90% of all the genes belonged to group B, and ~10% were in groups A or C at the 30- and 60-min time-points. The mean numbers (%) at 30 min were 1,611 (5%), 28,761 (90%) and 1,405 (4%) and at 60 min they were 1,324 (4.2%), 28,915 (91%) and 1,533 (5%) for groups A, B and C, respectively. Genes (n=19,070) (60% of the total) in all tumor samples from three cases and at all time-points belonged to group B. Of these, some housekeeping genes, such as GAPDH, actin β (ACTB), β-2-microglobulin (B2M), and 18S ribosomal RNA, showed high stable expression patterns (Fig. 2E). In contrast, none of the genes commonly belonged to group A, and only DDB1 and CUL4 associated factor 8-like 2 (DCAF8L2) commonly belonged to group C at all time-points.

### Quantitative RT-PCR detects random expression alterations

**Figure 3:**
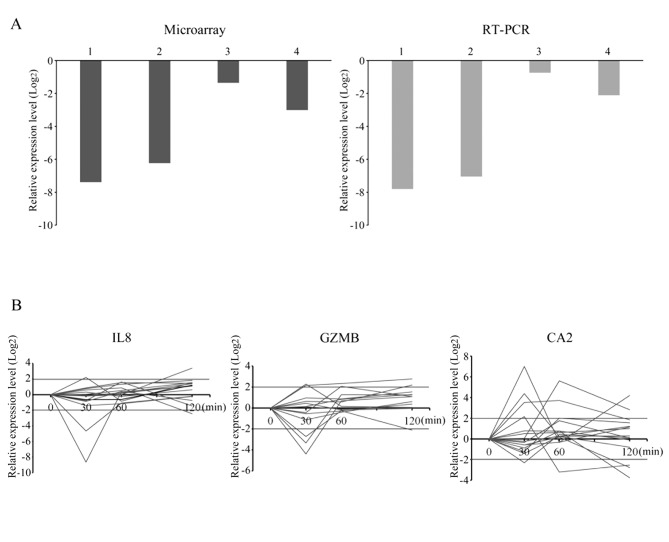
qRT-PCR analyses of gene expression levels in resected colorectal tumor samples. (A) Comparison of REG3A expression level between the microarray data and qRT-PCR in independent tumor samples. (B) qRT-PCR analyses for the colorectal cancer-associated genes IL8, GZMB and CA2 at all time points in 15 tumor cases.

To validate the microarray results, we performed qRT-PCR of REG3A, a gene that belonged to group C at the 120-min time-point. The expression levels of REG3A determined using qRT-PCR agreed with the microarray data in tumor samples (Fig. 3A). Furthermore, we investigated gene expression changes in IL8, GZMB and CA2, which have been reported to be associated with colorectal cancer progression, at all time-points in the 15 tumor cases (Fig. 3B). The expression levels of these genes were observed from the 30-min time-point, and the pattern of changes appeared to be incoherent.

### Bioinformatics clustering analyses of gene profiling fail to predict cases

**Figure 4:**
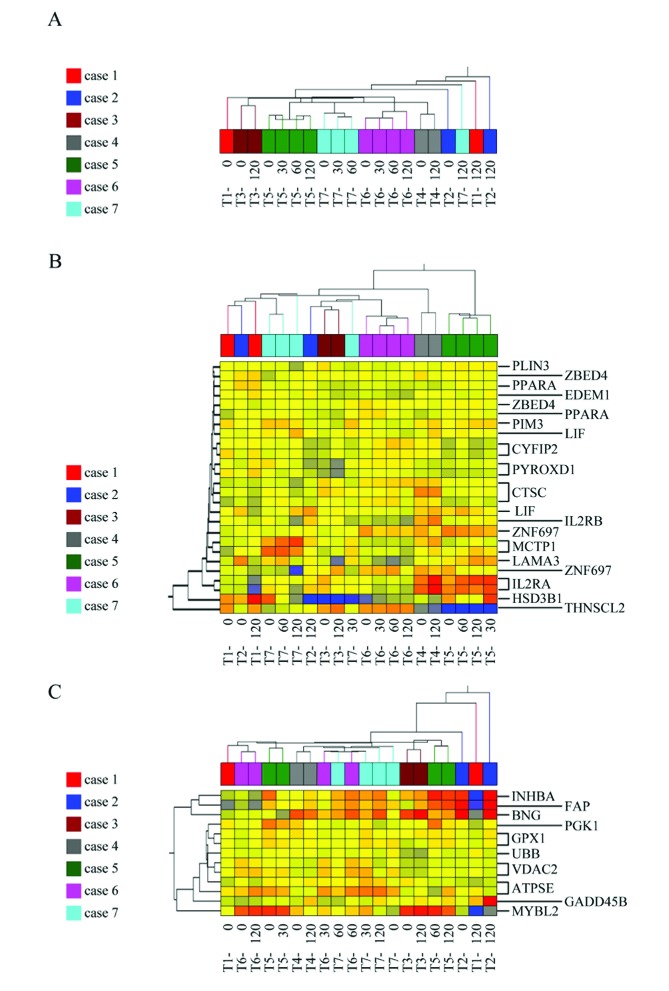
Bioinformatics hierarchical clustering analyses of gene profiling in seven resected colorectal cases. (A) Clustering analyses of all tumor samples using all the genes in the microarray data. Note that the heatmap of all the genes is not shown. (B and C) Clustering analyses applied to the 20 tumor samples using sets of genes identified by (B) Oncotype and (C) ColoPrint. The samples are listed in columns, and the mRNAs are listed in rows. The samples from each case are indicated with different colors at the top of the array. T, tumor.

The results of these previous studies prompted us to investigate whether the alteration of gene expression might cause critical bias in the quantitative analyses. Clustering analyses was applied to tumor samples from seven cases and four time-points. The analyses demonstrated that computer-based bioinformatics was unable to classify the samples according to cases, even using all the genes on the array chip (Fig. 4A). These data indicate that alterations in gene expression patterns that depend on the time-point could be a potential confounding factor for analyses. Moreover, we applied clustering analyses to the same samples using sets of genes identified by Oncotype (Fig. 4B) and ColoPrint (Fig. 4C). The analysis showed that samples that originated from the same case were classified into different clusters, indicating that computer-based bioinformatics could not distinguish samples case-by-case, even when well-established gene sets were used, possibly due to a timing-related bias.

## Discussion

For quantitative analyses using surgically resected tissues, the most probable bias is RNA degradation. Previous studies have shown that tissue degradation was limited in all types of human tissue except colorectal cancer at room temperature (23,24). Additionally, other studies have found significant degradation by measuring the RIN of human colon and rectal tissue with increasing ischemia time (25–27). In the present study, the samples showed high RIN values (above 7), indicating high RNA integrity. The differences between our results and these earlier reports in terms of RNA integrity likely resulted from different tissue manipulations (RNAlater reagent vs. liquid nitrogen). Other mechanisms that may decrease RNA integrity in colorectal tissue with increasing delay time could be the presence of normal gut flora, the increased rate of tissue turnover, and/or the presence of digestive enzymes in the gut (28). Using seven samples that had excellent RNA quality, we demonstrated that a set of genes could change expression levels among time-points, indicating that extraction timing is a crucial confounding factor for quantitative assessment of gene expression. We found that approximately 90% of genes did not change expression levels (within 2-fold) up to 120 min after surgical resection, which is in agreement with a report by Spruessel *et al* (11). Housekeeping genes such as GAPDH, 18S and ACTB were among the stably expressing genes across cases and time-points (group B) (Fig. 2B) and this is in accordance with previous results (29). This result is noteworthy as most qualitative experiments have been performed using these housekeeping genes as internal controls. Additionally, several colorectal biomarkers, including TS, DPD (18) and vascular endothelial growth factor (VEGF) (30) and most ColoPrint reference genes (8), including MCTP1, CTSC and PYROXD1, were in group B, indicating the reliability and reproducibility of these genes for molecular analyses in colorectal cancer. Of note, however, one ColoPrint-related gene, HSD3B1, had wide expression distribution among time-points. This is an alarming possible bias if it is used in quantitative analyses as it is a well-established biomarker. In contrast, 10% of genes belonged to groups A or C at each time-point in the present study. It has been reported that 15–30 min after surgery, 10–20% of genes differed significantly from baseline values (11). We suggest that these gene expression changes in the tumor are related to activity in response to ischemia, perhaps to allow the tumor cells to survive and participate in many complicated signaling pathways. Additionally, there is increasing awareness that colorectal tumors have to survive and grow in a poor microenvironment with low oxygen and glucose availability due to inadequacies of the tumor-associated vasculature (31,32). Musella *et al* (26) described that the genes that changed at 180 min in tumor samples were all oncogenes, and the expression levels over time increased.

Regarding the result of the qRT-PCR analysis, IL8, GZMB and CA2 constitute a set of specific gene-expression profiles that predict prognosis or recurrence. In the present study, we found that these biomarker genes had incoherently altered expression at all time-points, indicating the impact of ischemia time on changes in prognostic or predictive molecular biomarkers after surgical resection.

There are potential drawbacks to the present study. First, the potential for bias as a result of the surgical procedure remains. We used standard laparotomic or laparoscopic colorectal surgery that maintained tumor vascularity until just before surgical resection to minimize surgical stress, and we extracted the tissue samples at room temperature under clinical surgical conditions. Some studies have a profound clinical relevance as their results are subject to procedural effects rather than tumor biology (9). Second, considering the heterogeneity of tumorigenesis, we removed pieces of tumor tissue from the near part of the tumor lesion; however, the influence of inherent heterogeneity when using frozen tissue samples cannot be avoided (33).

Nevertheless, our data demonstrated the impact of timing on quantitative gene-expression profiles using the most robust microarray procedures. The impact of alterations of the gene expression pattern over time on biomarker analysis should be considered, as these time-dependent results could potentially mislead, and this could have immediate effects on decisions regarding patient treatment.

## Figures and Tables

**Table I:** Clinical and pathological characteristics of the 18 patients with colorectal cancer.

Characteristics	N
Gender	
Male	10
Female	8
Age (years)	
Mean (range)	72.4 (53–85)
Tumor location	
Right side	2
Left side	13
Rectum	3
Histologic type	
Well differentiated	9
Moderately differentiated	9
Others	0
Tumor size (mm)	
Mean (±SD)	47.8 (±15.0)
T status	
T1/T4	2
T2	13
T3	3
N status	
N0	10
N1/2	8
M status	
M0	18
M1	0
Stage	
I	0
II	10
III	8
IV	0
Surgical procedure	
Open	7
Laparoscopic	11
Blood loss (ml)	
Mean (±SD)	340.0 (±315.4)
Operative time (min)	
Mean (±SD)	278.8 (±111.5)
